# Benefit and predictive factors for speech perception outcomes in pediatric bilateral cochlear implant recipients^☆^

**DOI:** 10.1016/j.bjorl.2018.04.009

**Published:** 2018-05-18

**Authors:** Young-Soo Chang, Sung Hwa Hong, Eun Yeon Kim, Ji Eun Choi, Won-Ho Chung, Yang-Sun Cho, Il Joon Moon

**Affiliations:** a Sungkyunkwan University School of Medicine, Samsung Medical Center, Department of Otorhinolaryngology-Head and Neck Surgery, Seoul, South Korea; b Sungkyunkwan University School of Medicine, Samsung Changwon Hospital, Department of Otorhinolaryngology-Head and Neck Surgery, Seoul, South Korea; c Myongji University Graduate School, Department of Speech and Language Pathology, Seoul, South Korea; d Dankook University Hospital, Department of Otorhinolaryngology-Head and Neck Surgery, Cheonan, South Korea

**Keywords:** Bilateral cochlear implant, Pediatric, Predictive factors, Speech perception, Implante coclear bilateral, Pediátrico, Fatores preditivos, Percepção da fala

## Abstract

**Introduction:**

Despite recent advancement in the prediction of cochlear implant outcome, the benefit of bilateral procedures compared to bimodal stimulation and how we predict speech perception outcomes of sequential bilateral cochlear implant based on bimodal auditory performance in children remain unclear.

**Objectives:**

This investigation was performed: (1) to determine the benefit of sequential bilateral cochlear implant and (2) to identify the associated factors for the outcome of sequential bilateral cochlear implant.

**Methods:**

Observational and retrospective study. We retrospectively analyzed 29 patients with sequential cochlear implant following bimodal-fitting condition. Audiological evaluations were performed; the categories of auditory performance scores, speech perception with monosyllable and disyllables words, and the Korean version of Ling. Audiological evaluations were performed before sequential cochlear implant with the bimodal fitting condition (CI1 + HA) and one year after the sequential cochlear implant with bilateral cochlear implant condition (CI1 + CI2). The good performance group (GP) was defined as follows; 90% or higher in monosyllable and bisyllable tests with auditory-only condition or 20% or higher improvement of the scores with CI1 + CI2. Age at first implantation, inter-implant interval, categories of auditory performance score, and various comorbidities were analyzed by logistic regression analysis.

**Results:**

Compared to the CI1 + HA, CI1 + CI2 provided significant benefit in categories of auditory performance, speech perception, and Korean version of Ling results. Preoperative categories of auditory performance scores were the only associated factor for being GP (odds ratio = 4.38, 95% confidence interval – 95% = 1.07–17.93, *p* = 0.04).

**Conclusions:**

The children with limited language development in bimodal condition should be considered as the sequential bilateral cochlear implant and preoperative categories of auditory performance score could be used as the predictor in speech perception after sequential cochlear implant.

## Resumo

**Introdução:**

Apesar dos avanços recentes na predição do resultado do implante coclear, ainda não está claro o benefício do procedimento bilateral em comparação com a estimulação bimodal e como podemos prever resultados de percepção da fala com implante coclear bilateral sequencial com base no desempenho auditivo bimodal em crianças.

**Objetivos:**

Este estudo foi realizado para: 1) Determinar o benefício do implante coclear bilateral sequencial e 2) Identificar os fatores associados com o resultado do implante bilateral sequencial.

**Método:**

Estudo observacional e retrospectivo. Analisamos retrospectivamente 29 pacientes com implantes sequenciais após a adaptação bimodal. Avaliações audiológicas foram realizadas; os escores das categorias de desempenho auditivo, a percepção da fala com palavras monossílabas e dissílabas e a versão coreana de Ling. As avaliações audiológicas foram realizadas antes do implante sequencial com o ajuste bimodal (IC1 + AH) e um ano após o implante coclear sequencial com implante bilateral (IC1 + IC2). O grupo com bom desempenho (BD) foi definido da seguinte forma: 90% ou mais em testes com monossílabos e dissílabos com condição apenas auditiva ou melhoria de 20% ou mais dos escores com IC1 + IC2. A idade no primeiro implante, o intervalo interimplante, o escore categorias de desempenho auditivo e as diversas comorbidades foram analisadas através de análise de regressão logística.

**Resultados:**

Em comparação com o IC1 + AA, IC1 + IC2 demonstraram benefícios significativos nos escores categorias de desempenho auditivo, percepção da fala e a versão coreana de Ling. Os escores de categorias de desempenho auditivo pré-operatórios foram o único fator associado para ser do grupo BD (*odds ratio* - OR = 4,38, intervalo de confiança de 95% - IC 95% = 1,07-17,93, *p* = 0,04).

**Conclusões:**

As crianças com desenvolvimento limitado de linguagem em condição bimodal devem ser consideradas, pois o implante coclear bilateral sequencial e o escore pré-operatório das categorias de desempenho auditivo poderiam ser usados como preditores na percepção da fala após implante sequencial.

## Introduction

Surgical indications for Cochlear Implantation (CI) have been continuously expanded since the development of CI in the 1980s. At first, unilateral implantation was considered as the standard strategy for children with a bilateral profound hearing loss. Children with unilateral Cochlear Implants (CIs) have shown excellent speech perception abilities in controlled listening environments such as a quiet room or a sound-proof booth.^1^

However, developmental delays regarding speech/language in children with unilateral CI have been maintained or increased through adulthood.^2^, ^3^ Thus, a variety of strategies has been attempted to overcome this limitation and achieve a better outcome for unilateral CI users.

In recent years, several studies have shown the benefits of bimodal rehabilitation^4^ or bilateral CI^5^, ^6^ compared to monaural listening with only one CI.^7^ Therefore, bimodal fitting and bilateral CI are now accepted as safe and effective methods with bilateral auditory stimulation to achieve better speech/language outcome.^8^

However, if a contralateral residual hearing is insufficient and acoustic amplification of the non-implanted ear does not provide any benefit, sequential bilateral CI can be an alternative option to obtain better outcome.^9^ Language development may be limited even with the bimodal condition in certain patients. In these cases, sequential bilateral CI should be considered and the precise prediction of the performance outcome may be needed to perform a sequential CI. However, despite recent advancement in the prediction of CI outcome, the benefit of bilateral CI compared to bimodal stimulation and how we predict speech perception outcomes of sequential bilateral CI based on bimodal auditory performance in children remain unclear.

Therefore, the aims of the present study were: (1) to determine the benefit of sequential bilateral CI compared to bimodal fitting, and (2) to identify factors associated with the outcome of sequential bilateral CI in pediatric unilateral CI recipients.

## Methods

### Patients

We retrospectively reviewed a cohort of pediatric sequential bilateral CI recipients in one tertiary hospital from January 1, 2003 to December 31, 2012. Simultaneously implanted patients were excluded. Twenty-nine pediatric recipients (16 boys and 13 girls) performed sequential CI following bimodal fitting condition were enrolled in this study. All included children were prelingually deaf and sequentially implanted under bimodal-fitting condition. This study was approved by Institutional Review Board (2014, 910-083). All subjects gave written informed consent and assent forms prior to the beginning of testing.

In our center, the side of the first implantation was determined by the CI team considering hearing status, anomaly of inner ear structures, and visibility of the cochlear nerve on magnetic resonance imaging. Typically, the first implantation was performed at the worse-hearing side and a hearing aid was applied to the better-hearing side immediately after the diagnosis of pre-lingual deaf. Among these unilateral CI recipients with bimodal fitting and continuously using their bimodal fitting condition, those who did not show significant benefit from contralateral hearing aids during follow-up evaluations and did not reach their linguistic abilities of the children with unilateral CI on repetitive audiological/linguistic-verbal tests were considered as proper candidates for sequential bilateral CI recipients. Therefore, a second implant was sequentially implanted.

All patients were followed up for at least one year following bilateral implantation. They complied with our post-implant protocol, including regular speech therapy, consistent use of implants, and regular follow-up evaluations. All patients were born with profound hearing loss on both sides of the ear. Age at the first CI (CI1) ranged from 14 to 136 months (mean, 32.2 months; Standard Deviation (SD), 26.5 months). Age at the second CI (CI2) ranged from 38 to 176 months (mean, 72.3 months; SD, 36.4 months) (Table 1). The mean inter-implant interval was 40.1 months (SD, 18.0 months). Nine of the 29 children had several types of inner ear anomalies. Three of the 29 children showed associated developmental disorders such as global developmental delay, mental retardation, and Noonan syndrome (Supplementary Table 1). Inner ear anomalies were evaluated by preoperative computed tomography or magnetic resonance imaging. In all children, CI was successfully performed without any intraoperative complications.

**Table 1:** Subject demographics (*n* = 29).

Gender	M: 16/F: 13
CI1 ImpSide	R: 24/L: 5
Inner ear anomalies	None: 20/anomalies: 9
Combined with developmental disorder	None: 26/disorder: 3

	Mean	SD	Median
Age at CI1 (mos)	32.2	26.5	23.0
Age at CI2 (mos)	72.3	36.4	60.0
Inter-implant interval (mos)	40.1	18.0	35.0
CI1 + HA CAP	4.7	1.7	5.0

M, Male; F, Female; R, Right; L, Left; ImpSide, Side of ear with an implant; CI1, First Cochlear Implant; CAP, Categories of Auditory Performance scale; mos, months; CI1 + HA, the audiological evaluations performed with the bimodal fitting condition before sequential cochlear implant.

### Measures

Preoperative CI work-up protocol for children included audiological, developmental, psychological, neurological, and ophthalmologic evaluations with routine tests for general anesthesia. Pre- and post-operative audiological evaluations were performed. The auditory perception was evaluated with Categories of Auditory Performance (CAP) scores. The CAP score evaluation processed blindly by an Experienced Speech Therapist (EYK). Speech perception in both Auditory-Only (AO) and Audiovisual (AV) conditions were evaluated with monosyllable and bisyllable words. Stimuli were presented using a monitored live voice of a clinician whose face was hidden behind a mesh screen. The presentation level was approximately 70 dB SPL determined with a handheld sound level meter as previously described.^10^ Patients were instructed to repeat each stimulus item presented. The Experienced Speech Therapist (EYK) was present during the testing to transcribe patient verbal responses. The stimulus items used in each test were presented in a random order. Speech production and language ability was assessed with Korean Version of the Ling's Stage (K-Ling).^11^ A questionnaire for evaluating the satisfaction by each parent was asked at one year after performing the sequential bilateral CI and compared to the satisfaction with bimodal CI. Subjective satisfaction was rated as +5 (very satisfied) to −5 (very disappointed). The questionnaire used in this study is shown in Table 2.

**Table 2:** The questionnaires.

Question 1 (General satisfaction)	How much do your child generally satisfied with bilateral cochlear implantation compared with one side cochlear implant and one side hearing aid?
Question 2 (Sound localization)	How much do your child satisfied with recognizing the sound direction in daily life with bilateral cochlear implantation compared with one side cochlear implant and one side hearing aid?
Question 3 (Word recognition in noise)	How much do your child satisfied with understating the word in noisy conditions with bilateral cochlear implantation compared with one side cochlear implant and one side hearing aid?

### Test protocols

One to three months before the sequential CI, audiological evaluations were performed with bimodal fitting condition (CI1 + HA). Bilateral CI performance (CI1 + CI2) was evaluated at one year after the sequential second implant.

At first, we compared the audiological test results between bimodal and bilateral conditions to identify the audiological benefit of sequential bilateral CI. The bimodal condition was tested before bilateral implantation. The bilateral condition was tested at one-year following the second implant. Questionnaire results were analyzed to identify the benefit of sequential bilateral CI.

To evaluate the outcome of speech perception, we analyzed the outcomes into a divided group according to the performance: Good Performer Group (GP) and a Poor Performer Group (PP). GP was defined as follows: ≥90% in monosyllable and bisyllable tests with AO condition or ≥20% improvement in the scores with sequential bilateral CI when compared to those obtained in bimodal conditions.

We then identified factors associated with the outcomes of sequential bilateral CI (CI1 + CI2), including age at first implantation, inter-implant interval, CAP score evaluated with the bimodal condition before the second CI, and various comorbidities of patients. According to comorbidities, patients were assigned into the following groups: (1) Without any comorbidities; (2) With inner ear anomalies but without any associated developmental disorders; and (3) With associated developmental disorders regardless of inner ear anomalies. After analyzing the benefit and factors association with sequential bilateral CI, we analyzed the basic characteristics of patients who did not reach the criteria of GP.

### Statistical methods

Quantitative variables were summarized in means (SD) and/or medians. Categorical variables were summarized in frequencies. Changes in mean performance scores were analyzed using paired *t*-tests or Wilcoxon signed rank tests. The association between factors and outcomes of sequential bilateral CI were examined using logistic regression analysis. All analyses were performed with the Statistical Package for Social Science version 20 (SPSS, Chicago, IL, USA). A *p*-value ≤ 0.05 was considered as statistically significant.

## Results

### Benefit of sequential bilateral CI compared to bimodal fitting

All performance results are summarized in Table 3. Compared to bimodal scores (CI1 + HA), sequential bilateral CI provided significantly better audiological benefit based on CAP scores. The average CAP score with the bimodal condition was 4.7. It was increased (*p* < 0.001, paired *t*-test) to 5.7 with the sequential bilateral CI. The difference in CAP score between the two conditions was 1.00 (SD ± 1.31).

**Table 3:** Mean performance scores.

		N°	Mean (%)	SD	Median
CI1 + HA	CAP	29	4.71	1.72	5.0
	AO-m (%)	29	47.3	40.4	60.0
	AO-bi (%)	29	52.6	43.7	76.0
	AV-m (%)	29	57.5	46.8	84.0
	AV-bi (%)	29	59.7	48.0	96.0
	K-Ling phonologic level	29	5.4	2.1	6.0
	K-Ling phonetic level	29	4.9	2.0	5.0

CI1 + CI2	CAP	28	5.71	1.12	6.0
	AO-m (%)	29	76.1	34.1	90.0
	AO-bi (%)	29	79.7	35.0	96.0
	AV-m (%)	29	84.1	34.9	100.0
	AV-bi (%)	29	84.8	34.2	100.0
	K-Ling phonologic level	29	6.5	1.9	7.0
	K-Ling phonetic level	29	6.2	1.8	7.0

CI1 + HA, The audiological evaluations performed with the bimodal fitting condition before sequential cochlear implant; CAP, Categories of Auditory Performance scale; AO, Auditory-Only modes; AV, Audiovisual Modes; m, Monosyllable recognition; bi, Bisyllable recognition; K-Ling phonologic level, Korean Version of the Ling's Stage phonologic level; Pre-CI2 K-Ling phonetic level, Korean Version of the Ling's Stage phonetic level; CI1 + CI2, The audiological evaluations performed with the bilateral cochlear implant one year after the sequential second implant.

**Figure 1:**
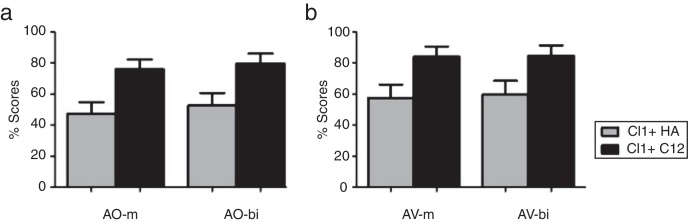
Auditory-only (a) and audiovisual (b) speech perception performance with CI1 + HA and CI1 + CI2 (m, monosyllable recognition; bi, bisyllable recognition).

Both phonologic level and phonetic level were significantly improved with sequential bilateral CI. The mean K-Ling phonologic level was significantly improved from bimodal to sequential bilateral CI (5.4–6.5, *p* < 0.001, Wilcoxon signed rank test). The difference in K-Ling phonologic level between the two conditions was 1.07 (SD ± 1.13). The mean K-Ling phonetic level was also significantly improved from bimodal condition to sequential bilateral CI (4.9–6.2, *p* < 0.001, Wilcoxon signed rank test). The difference in K-Ling phonetic level between the two conditions was 1.34 (SD ± 1.17). Benefit in speech perception performance (monosyllable and disyllables) was also found to be significantly improved from bimodal condition to sequential bilateral CI in both AO mode (monosyllable: 47.3–76.1%, *p* < 0.001; disyllable: 52.6–79.7%, *p* < 0.001, respectively) and AV mode (monosyllable: 52.6–84.1%, *p* < 0.001; bisyllable: 57.5–84.8%, *p* < 0.001, respectively) (Fig. 1).

**Figure 2:**
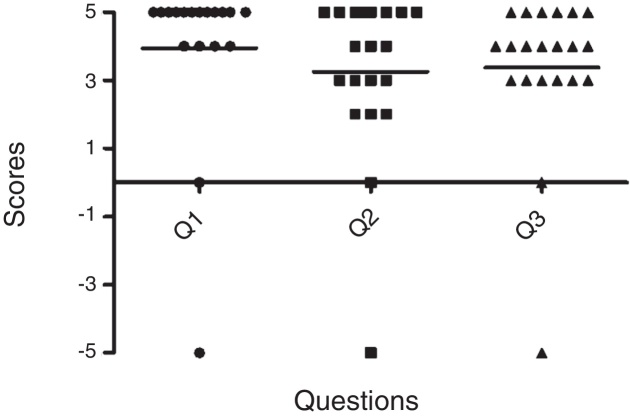
Significant outcome of satisfaction observed in parents of 22 (75.9%) out of 29 children (Q1, general satisfaction; Q2, sound localization, Q3, word recognition in noise).

Parents of 22 (75.9%) out of 29 children answered the questionnaires. General satisfaction score, sound localization score, and word recognition in noise score were 3.23 (SD ± 2.76), 2.95 (SD ± 2.48), 3.23 (SD ± 2.31), respectively (Fig. 2). The mean rank of each questionnaire according to comorbidities did not show any significant difference (Kruskal–Wallis test). Only one child reported the discomfort with second CI. Despite the dizzy feeling suffered from second CI occasionally, the child showed an overall improvement in the audiological test with sequential bilateral CI.

### Factors associated with the outcome of sequential bilateral CI

**Figure 3:**
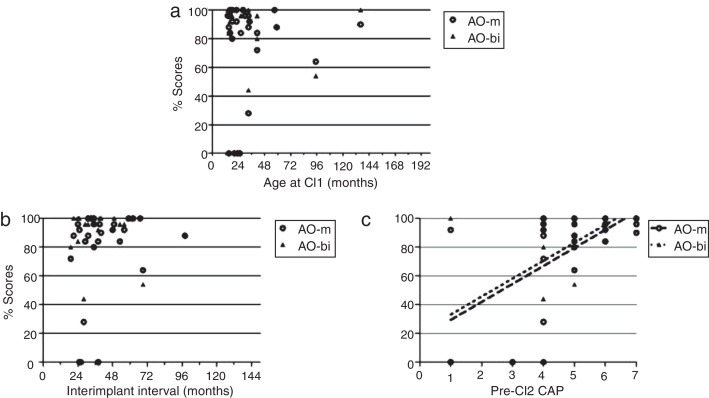
Scattergram of scores of AO-m (blank circle) and AO-bi (black triangle) in bilateral CI condition according to age at CI1 (a), inter-implant interval (b), and CI1 + HA CAP score (c). CI1, first cochlear implant; AO, auditory-only mode; AV, audiovisual mode; m, monosyllable recognition; bi, bisyllable recognition.

Using our criteria, 24 (82.8%) out of 29 children were found to have GP. However, five children failed to meet the GP criteria. Logistic regression analysis showed that CI1 + HA CAP score was the only significant factor associated with GP (OR = 4.38, 95%CI 1.07–17.93, *p* = 0.04). Other factors failed to reach statistical significance in the association with speech perception outcomes (Supplementary Table 2 and Fig. 3). The demographics of GP and PP group are summarized in Table 4.

**Table 4:** Demographics of good performer and poop performer group.

	Good performer (*n* = 24)	Poor performer (*n* = 5)	*p*-value
*Gender*			1.00^a^
Male	13	3	
Female	11	2	
*Inner ear anomalies*			0.29^a^
None	18	2	
Anomalies	6	3	
*Combined with developmental disorder*			1.00^a^
None	22	4	
Disorder	2	1	
*Age at CI1 (mos)*	33.04 ± 28.23	28.40 ± 17.52	0.93^b^
*Age at CI2 (mos)*	72.63 ± 34.55	71.00 ± 48.81	0.38^b^
*Inter-implant interval (mos)*	39.58 ± 14.86	42.60 ± 31.43	0.67^b^
*CI1* *+* *HA CAP*	5.13 ± 1.39	2.80 ± 1.79	0.01^b^

CI1 + HA, The audiological evaluations performed with the bimodal fitting condition before sequential cochlear implant; CAP, Categories of Auditory Performance scale; mos, months.

a Fisher's exact test.

b Mann–Whitney test.

## Discussion

The present study revealed that sequential bilateral CI could give more benefit to speech perception than bimodal condition when benefit from hearing aids was limited. The CAP score, K-Ling phonologic/phonetic level and speech perception evaluated by monosyllable and bisyllable in AO/AV modes showed significant improvement with sequential bilateral CI compared to those with the bimodal condition. This finding is similar to the result of Blamey^12^ indicating that a second CI is likely to provide slightly better postoperative speech outcome than an additional HA. Previous studies have reported the benefit of bilateral CI compared to bimodal rehabilitation in noise condition^13^ or in sound localization test. In addition to previous knowledge, we elucidated that sequential bilateral CI in a quite condition had speech perception benefit compared to bimodal rehabilitation. This study included children with inner ear anomalies or developmental disorder. The speech perception benefit was consistent in those children. Besides, sequential bilateral CI showed benefit in sound localization and word recognition in noise conditions after analyzing parents-reported child's satisfaction with sequential bilateral CI.

We administrated a criterion for GP for the evaluation of sequential bilateral CI and identified that better preoperative CAP score was significantly associated with GP following the second CI implantation. When we compared speech performance between bimodal stimulation and sequential bilateral CI, we adopted AO stimulation results. Verbal information transmitted to listeners via AV stimulation is often thought to be more efficient than AO stimulation.^14^ To minimize the ceiling effect of the test result, we introduced the result of AO stimulation to comparative analysis. Despite the ceiling effect in performance, this result can be used to predict the performance of 2nd CI with preoperative CAP score.

It has been reported that the benefits of sequential bilateral CI in the short term are the clearest in children with limited delays between the two sequential implantations.^15^ In our analysis, however, the inter-implant interval did not show any significant association with speech performance with sequential bilateral CI. This factor was eliminated during logistic regression analysis in this study. This finding might be related to the continuous stimulation in both sides of the ear with CI and HA. Although the residual hearing on the contralateral ear to the 1st CI side was minimal, our enrolled children had started to use HA on their contralateral ear after the diagnosis of profound hearing loss on both sides of the ear and kept using until the second implantation. It has been revealed that the development of auditory cortex is significantly influenced by continuous hearing stimulation even in congenitally deaf animals.^16^ Therefore, such uninterrupted auditory stimulation might have provided protection against the deprivation of auditory cortex or nerve in the non-CI side ear and minimalized the effect of the inter-implant interval. Therefore, our finding suggests that early administration of HA on the contralateral side to the 1st CI might be needed. In addition, recent studies reported that inter-implant interval was not correlated with performance measures in the adolescent population or in a younger group of children and these studies concluded that a second implant could be beneficial even after a substantial interval between implant.^17^

The present study aimed to determine the benefit of sequential bilateral CI compared to bimodal condition and identify factors associated with auditory performance with sequential bilateral CI in pediatric population. However, due to its retrospective nature, this study has some limitations. We evaluated speech perception in both AO and AV stimulation modes using monosyllable and bisyllable words. However, it has been reported the ceiling effect in this evaluation. In addition, a recent study suggested that the use of monitored live voice for the assessment of speech perception in the pediatric audiology clinic may overinflate children‘s performance.^18^ Both the ceiling effect and the overinflated performance following the use of monitored live voice could affect the clarification of children‘s performance. In addition, the possible influences of numerous factors may have been underestimated in this retrospective study. Especially when we interpreted the results with parents’ questionnaire analysis, we should keep in mind of possible bias with recollections. Furthermore, those children with good speech perception could have limited performance in real life conditions such as noise condition. To minimize the effect of these limitations, we surveyed parents-reported satisfaction on sequential bilateral CI and found that sequential bilateral CI had benefit in daily life. Further evaluation of speech in noise or sound localization performance in controlled conditions, which are usually carried out in a more realistic and challenging environment and might have benefit to evaluate the more accurate performance, is needed to elucidate the additional benefits and factors associated with sequential bilateral CI compared to bimodal conditions.

## Conclusion

In summary, results of this study showed that sequential bilateral CI had additional benefit compared to bimodal condition regarding CAP score, K-Ling phonologic/phonetic level, and speech perception level. Children with higher preoperative CAP score in bimodal conditions had significantly positive association with good performance in speech perception after sequential CI. Therefore, children with limited language development in bimodal condition should be considered as the sequential bilateral CI regardless of the inter-implant interval and preoperative CAP score could be used as the predictor of the good performance in speech perception after sequential CI.

## Ethical approval

This study was approved by Samsung Medical Center Institutional Review Board (2014-910-083).

## Conflicts of interest

The authors declare no conflicts of interest.
