# Supplementary material for: Benefit and predictive factors for speech perception outcomes in pediatric bilateral cochlear implant recipients
Source: Braz J Otorhinolaryngol. 2018 May 18;85(5):571–7. doi: 10.1016/j.bjorl.2018.04.009 (PMC9443016; doi:10.1016/j.bjorl.2018.04.009)
Supplement: Supplementary file 1 [file mmc1.doc]

**Supplementary Table 1** Distribution of additional anomalies of patients (n = 9).

| **Patient nº** | **Inner ear anomalies** | **Developmental disorder** | **Age at CI1 (mos)** | **CI1+HA CAP** |
| --- | --- | --- | --- | --- |
| 1 | Mondini dysplasia | Developmental delay | 33 | 7 |
| 2 | EVAS |  | 41 | 4 |
| 3 | EVAS |  | 18 | 5 |
| 4 |  | Mental retardation | 33 | 4 |
| 5 | EVAS  Poorly delineated CN |  | 23 | 1 |
| 6 | EVAS | Noonan syndrome | 18 | 6 |
| 7 | Mondini dysplasia |  | 25 | 4 |
| 8 | EVAS  Poorly delineated CN |  | 18 | 4 |
| 9 | Poorly delineated CN |  | 15 | 1 |

Nº, Number; CI1, First cochlear implant; EVAS, Enlarged Vestibular Aqueduct Syndrome; CN, Cochlear Nerve; CAP, Categories of Auditory Performance scale; mos, months; CI1+HA, The audiological evaluations performed with the bimodal fitting condition before sequential cochlear implant.

**Supplementary Table 2** Predictors of speech perception outcomes with sequential bilateral CI.

|  | **B** | **SE** | **p-value** | **OR** | **95% CI** |
| --- | --- | --- | --- | --- | --- |
| **Age at CI1** | 0.03 | 0.07 | 0.66 | 1.03 | 0.91–1.17 |
| **Inter-implant interval** | -0.11 | 0.06 | 0.08 | 0.89 | 0.79–1.01 |
| **CI1 + HA CAP** | 1.48 | 0.72 | 0.04 | 4.38 | 1.07–17.93 |
| **Comorbidity** |  |  | 0.32 |  |  |
| Inner ear anomalies | -3.03 | 2.08 | 0.13 | 0.05 | 0.01–2.85 |
| Developmental disorder | 16.84 | 22107.59 | 0.99 | ‒ | ‒ |
| **Constant** | 0.58 | 2.47 | 0.81 | 1.79 |  |

CI1, First Cochlear Implant; CI1+HA, The audiological evaluations performed with the bimodal fitting condition before sequential cochlear implant; CAP, Categories of Auditory Performance scale; B, Coefficient; SE, Standard Errors with coefficient; OR, Odds Ratio; CI, Confidence Intervals.
